# Seasonal Variation of Water Quality in Unregulated Domestic Wells

**DOI:** 10.3390/ijerph16091569

**Published:** 2019-05-05

**Authors:** Yoshira Ornelas Van Horne, Jennifer Parks, Thien Tran, Leif Abrell, Kelly A. Reynolds, Paloma I. Beamer

**Affiliations:** 1 Mel and Enid Zuckerman College of Public Health, University of Arizona, 1295 N. Martin Ave., Tucson, AZ 85724, USA; reynolds@email.arizona.edu (K.A.R.); pbeamer@email.arizona.edu (P.I.B.); 2 Friends of the Santa Cruz River, P.O. Box 4275, Tubac, AZ 85646, USA; j_parks78@msn.com; 3 Department of Soil, Water & Environmental Science, University of Arizona, Tucson, AZ 85721-0038, USA; teetranuofa1992@gmail.com (T.T.); abrell@u.arizona.edu (L.A.); 4 Department of Chemistry and Biochemistry, University of Arizona, Tucson, AZ 85721-0041, USA

**Keywords:** arsenic, *Escherichia coli*, PFOA, PFOS, private well water, sucralose, water quality

## Abstract

In the United States (U.S.), up to 14% of the population depend on private wells as their primary drinking water source. The U.S. government does not regulate contaminants in private wells. The goals of this study were to investigate the quality of drinking water from unregulated private wells within one mile (1.6 kilometers) of an effluent-dominated river in the arid Southwest, determine differences in contaminant levels between wet and dry seasons, and identify contributions from human sources by specifically measuring man-made organic contaminants (perfluorooctanoic acid (PFOA), perfluorooctane sulfate (PFOS), and sucralose). Samples were collected during two dry seasons and two wet seasons over the course of two years and analyzed for microbial (*Escherichia coli*), inorganic (arsenic, cadmium, chromium, copper, lead, mercury, nitrate), and synthetic organic (PFOA, PFOS, and sucralose) contaminants. Arsenic, nitrate, and *Escherichia coli* concentrations exceeded their respective regulatory levels of 0.01 mg/L, 10 mg/L, and 1 colony forming unit (CFU)/100 mL, respectively. The measured concentrations of PFOA and PFOS exceeded the respective Public Health Advisory level. Arsenic, PFOA, PFOS, and sucralose were significantly higher during the dry seasons, whereas *E. coli* was higher during the wet seasons. While some contaminants were correlated (e.g., As and Hg ρ = 0.87; PFOA and PFOS ρ = 0.45), the lack of correlation between different contaminant types indicates that they may arise from different sources. Multi-faceted interventions are needed to reduce exposure to drinking water above health-based guidelines.

## 1. Introduction

Up to 45 million people (14% of the total population) in the United States (U.S.) rely on private wells as their primary drinking water source [1]. Drinking water regulations do not apply to private wells and lack of regular monitoring in the U.S. poses a potential public health risk [2,3,4]. Although drinking water regulations do not apply to private wells, the population that relies on private well water may be drinking water with contaminants at concentrations exceeding the U.S. Environmental Protection Agency’s (EPA) Maximum Contaminant Levels (MCL). Although they do not apply to privately owned well water systems, it is recommended that well users not drink water above these health-based standards. It is not clear to what degree the individuals accessing these water sources are aware of the potential public health risks associated with private well water sources [5,6,7].

Studies have reported contamination of private well water with inorganic (e.g., arsenic, cadmium, chromium, copper, lead, mercury, and nitrate), bacterial (e.g., *Escherichia coli* (*E. coli*), and synthetic organic compounds (e.g., perfluorooctanoic acid (PFOA), perfluorooctane sulfate (PFOS), sucralose) [2,3,8,9,10,11,12,13,14,15,16,17,18,19,20]. The presence of these contaminants in private well water can stem from proximity to septic tanks, plumbing, sanitary sewer overflows, natural geology, agricultural and urban storm water runoff, and industrial processes [2,17,21,22,23,24]. While the inorganic and microbial contaminants reported in this study have been reported previously in a nationwide study, it is important to note that they did not investigate PFOS, PFOA, or sucralose [9]. Research is needed to determine if measuring these contaminants together can help increase the understanding of their primary contaminant sources in order to reduce exposures and potential health risk to private well water users. 

A majority of the water flow in effluent-dominated rivers can be attributed to discharge from industrial and/or municipal wastewater treatment plants [25,26,27]. In arid or semi-arid regions, stream flows can be comprised of more than 90% effluent [26]. The impact of wastewater effluent on groundwater quality has been documented, with studies reporting that shallow, unconfined aquifers are vulnerable to surface water contamination [28,29,30]. Pumping groundwater in close proximity to surface water, such as by private wells, may increase the exchange between groundwater and surface water [31]. In Santa Cruz County, Arizona, the cities of Nogales, Rio Rico, and Tubac rely primarily on groundwater for their drinking water supply through public utilities and privately owned wells [32]. Effluent discharged from the Nogales International Wastewater Treatment Plant (NIWTP) serving both the U.S and Mexico [25,33], sanitary sewer overflows [24], proximity to farmland [12], and industrial businesses [21] may potentially contribute to the contaminants measured in the Santa Cruz River. Water drawn from private wells may be contaminated due to their close proximity to the Santa Cruz River and their downgradient location in relation to the NIWTP.

The primary objective of this research was to characterize contamination patterns in private wells within a one mile (1.6 km) distance of the Santa Cruz River and examine if there was a difference in contaminant levels by season (dry vs. wet). Examining the contaminant levels by season provides insights regarding the influence of heavy precipitation events (i.e., monsoon) on effluent and runoff intrusion into private well water used for drinking. Measurement of both man-made contaminants (PFOS, PFOA, and sucralose) and those from potentially naturally occurring sources (As, Cd, Cr, Cu, Hg, Pb, and *E. coli*) provides a unique opportunity to understand potential contamination patterns.

## 2. Materials and Methods 

### 2.1. Recruitment

This study was a partnership between the non-profit organization Friends of the Santa Cruz River (FOSCR) and the University of Arizona. Together funding was secured from the U.S. EPA’s Environmental Justice Small Grants Program. This study was reviewed and approved by the University of Arizona Institutional Review Board (protocol number 1200000059). Eligible participants were required to be connected to a private-domestic well within one mile (1.6 km) of either the Nogales Wash or the Santa Cruz River, reside in Santa Cruz County, AZ, and use this water source as their primary drinking water supply. This is a convenience sample and well owners were recruited for participation in the study via a mail invitation or a phone call. Contact information for owners with a private well within one mile (1.6 km) of the river or the wash was obtained from the Arizona Department of Water Resources, the Friends of the Santa Cruz River, and the Arizona Department of Environmental Quality well registries. Participants who agreed to participate provided written informed consent. 

### 2.2. Determination of Wet and Dry Seasons

Historical rain data was obtained from the Arizona Department of Water Resources and used to determine seasonal periods of heavy precipitation (Table 1). 

In Arizona, the dry season has historically occurred in the months of April, May, and June, while the wet season has occurred in July and August. This wet season is the Arizona monsoon season, where on average at least half of the annual rainfall is received during the two-month period (Table 1). For this project, dry season sampling occurred during June and July for both 2013 and 2014. Historically, July has been considered part of the wet season, but the sampling area did not receive rain in early July 2013 and 2014. The wet season samples were collected during late July and August of 2013 and 2014.

### 2.3. Sample Collection 

A total of 80 tap water samples from 22 private wells were collected over a 15-month period. Each well was sampled four times, twice in the dry season (June and/or July 2013 and 2014), and twice in the wet season (July and/or August 2013 and 2014). In the first year, there were 22 wells enrolled, but four dropped out in the second year. The 22 wells were all located between Tubac, AZ, and Nogales, AZ, which is about a 20-mile (32.2-km) strip. Total dissolved solids, pH, conductivity, and temperature were measured at the time of sample collection using a Beckman Coulter 400 series handheld meter (Pasadena, CA, USA). At each home, the kitchen sink faucet was cleaned using a 70% ethanol solution and paper towels. Following the U.S. EPA’s drinking water collection methods, powder free nitrile disposable gloves were worn and the faucet was flushed for 2 minutes before collection of water samples [34]. Most (76/80) well water samples were collected from the kitchen sink. There were four well water samples collected from the wellhead and not the kitchen sink. At each visit, a 1-L water sample was collected in a polypropylene plastic container containing sodium thiosulfate for *E. coli* analysis, a 50-mL sample was collected in a propylene tube for analysis of inorganic contaminants (As, Cd, Cr, Cu, Pub, Hg, NO_3_^−^), and an additional 1-L sample was taken in an amber glass bottle (pre-cleaned and baked for 4 hours at 475 °C) for analysis of organic contaminants (PFOS, PFOA, sucralose). For *E. coli* and inorganic contaminants, ASTM Type I water blank controls were taken into the field and treated the same as samples. For organic analyses, a water blank control sample was collected with every third or fourth sample by transferring 1 L of an unopened bottle of LCMS grade H_2_O (J.T Baker) into one of the baked 1-L amber bottles. The containers were placed in a cooler and kept on ice until they were delivered to the laboratories.

### 2.4. Analysis of Microbial Contaminants 

Samples were analyzed within 24 hours of collection for *E. coli*. A 100-mL aliquot of the sample was filtered through a membrane (0.45 μm) and the filter was plated on m-Endo Agar LES (Becton Dickinson and Company, Sparks, MD, USA). A total of three replicate filtrations occurred for each sample. After 24 hours of being plated on the m-ENDO media, total colonies growing on the plate were counted and recorded. The filter membrane was then transferred onto Nutrient Agar with MUG plates (Becton Dickinson and Company Sparks, MD, USA). After 4 hours of incubation, Nutrient Agar with MUG plates were observed under UV light for fluorescence. Fluorescence of colonies confirmed the colony as *E. coli*. During analysis, a negative control consisting of *Klebsiella* and a positive control of *E. coli* were utilized for quality control and quality assurance [35]. The limit of detection for *E. coli* was 0.3 colony forming unit (CFU)/100 mL. 

### 2.5. Analysis of Inorganic Contaminants 

Inorganic analyses, except for nitrate, were performed by inductively coupled plasma-mass spectrometry (ICP-MS, Elan DRC-II) with a High-Performance Liquid Chromatography system according to U.S. EPA Method 200.8 (Perkin Elmer Series 200 HPLC system, Hopkinton, MA, USA). The limits of detection (LODs) were as follows: arsenic (1.6 × 10^−5^ mg/L), cadmium (2 × 10^−6^ mg/L), chromium (1.4 × 10^−5^ mg/L), copper (3.2 × 10^−5^ mg/L), lead (2 × 10^−7^ mg/L), and mercury (5 × 10^−6^ mg/L). Nitrate was analyzed by ion chromatography (Perkin Elmer Series 200) and measured as nitrogen (LOD = 1 × 10^−5^ mg/L) according to U.S. EPA Method 300.1. All inorganic analyses were conducted at the Arizona Laboratory of Emerging Contaminants (ALEC).

### 2.6. Analysis of Organic Contaminants

Water samples for organic contaminants analyses were solid phase extracted (SPE) using a Caliper Life Sciences AutoTrace device and Oasis HLB cartridges 6cc (150 mg; Waters Corp., Milford, MA, USA), with 0.5 g of ethylenediaminetetraacetic acid (EDTA) (added to chelate natural free metal ions that can potentially cause problems for extraction and analysis) and the following internal standards: ^13^C-labeled PFOA and PFOS (Wellington Laboratories, Guelph, ON, Canada) and Sucralose-d_6_ (Toronto Research Chemicals Inc., North York, ON, Canada) each added to a concentration of ca. 0.8 ppm. SPE proceeded as follows: cartridges were conditioned with 5.0 mL of each solvent in the following order: methyl tertiary butyl ether (MTBE), methanol, and LCMS water; 1 L of water was loaded at 10 mL/min and dried under nitrogen for 40 minutes; samples were eluted with 3.0 mL methanol, 3.0 mL 5% NH_4_OAc in MeOH, 3.0 mL acetonitrile, and 3.0 mL MTBE. Extracts were concentrated and then transferred to 2.0-mL autosampler vials for measurement by Liquid Chromatography Tandem Mass Spectrometry (LC-MS/MS, Dionex Ultimate 3000 RSLC UPLC, Waltham, MA, USA with Sciex TripleTOF 5600, Framingham, MA, USA). Samples (10 μL injections) were separated on a Zorbax Eclipse Plus C18 (2.1 × 50 mm, 1.8 uM; Agilent Technologies, Santa Clara, CA, USA) column with a reversed-phase gradient (A: 2 mM NH_4_OAC in MeOH, and B: 2 mM NH_4_OAc in LCMS-H_2_O:MeOH (95:5)) at 0.35 mL min^−1^ as follows: 0–0.5 min 25% B, 0.5–5.0 min increasing to 85% B, 5.0–5.1 min increasing to 100% B, 5.1–8.8 min held at 100% B, returning to 25% B at 9.0 min for re-equilibration. Target analytes were ionized by negative mode electrospray ionization at 4.5 kV with a declustering potential of −80 V and source temperature of 700 ºC with nebulizer, heater, and curtain gases at 55, 55, and 30, respectively. Precursor ions were selected for product ion measurement (using a mass range of 0.01 Da) with collision energies (CE) as follows: PFOA 413.0 > 368.9507, CE −15; PFOS 498.9 > 79.9751, CE −50; sucralose measured as a molecular ion at 397.0031. Analyst TF 1.6.1 and MultiQuant 2.1 (Sciex, Framingham, MA, USA) software programs were used to quantify target analytes with calibration curves and accounting for internal standard measurements. Method LODs (mg/L) were as follows (in dry and wet seasons, respectively): sucralose (6.44 × 10^−6^, 3.87 × 10^−6^), PFOS (3.44 × 10^−6^, 8.44 × 10^−7^), PFOA (2.49 × 10^−5^, 3.64 × 10^−6^). All organic analyses were conducted at ALEC.

### 2.7. Data Analysis 

Samples were blank corrected and inorganic and organic contaminant concentrations reported as non-detectable were substituted with the limit of detection divided by the square root of two [36]. Samples with no *E. coli* detected were substituted with the LOD [37]. All blanks were below the limit of detection. A total of 80 samples collected over a 15-month period were available for analysis (over the course of the study, four well owners dropped out). All statistical analyses were completed using STATA 12.0 (StataCorp, College Station, TX, USA). Concentration differences by season (dry vs. wet) of inorganic and organic contaminants were assessed using Wilcoxon signed-rank tests. For contaminants with a detection frequency less than 50 % (*E. coli*, Cd, PFOA, and sucralose), McNemar’s test was used to test for detection frequencies by season. Additionally, Spearman pairwise rank correlation was used to identify correlations between inorganic, organic, and microbial contaminants.

## 3. Results

### 3.1. Sampled Population Characteristcs 

The study population was composed of 64% males and 36% females. Half of the participants self-reported as White, with 45.5% self-reporting as Hispanic. Additionally, 64% reported to be in the 50–59 age range, 59.1% had a college degree, 73% had been living in their current home for over 10 years, and 59.1% reported a household annual income above $30,000 USD. The demographics of the study participants differ from those that reside in Santa Cruz County, AZ. As of the 2010 census, residents of Santa Cruz County were 48.1% males and 51.9% females, with the median age reported as 35.6 years of age. The racial makeup reported was 75.3% White, with 82.8% self-reporting as Hispanic. Additionally, 22.1% had a college degree, and the median household income was $39,630 USD. While comparison to only well-water owners is not possible, census data for the area sampled indicates that our participants are older, are more likely to have a college degree, and more likely to be male.

### 3.2. Water Quality Parameters 

The in-field water quality parameters of temperature, pH, conductivity, and total dissolved solids (TDS) were not statically different between seasons (Table 2). TDS and pH met the U.S. National Secondary Drinking Water Regulations.

### 3.3. Summary of Contaminants in Water Samples

**Figure 1:**
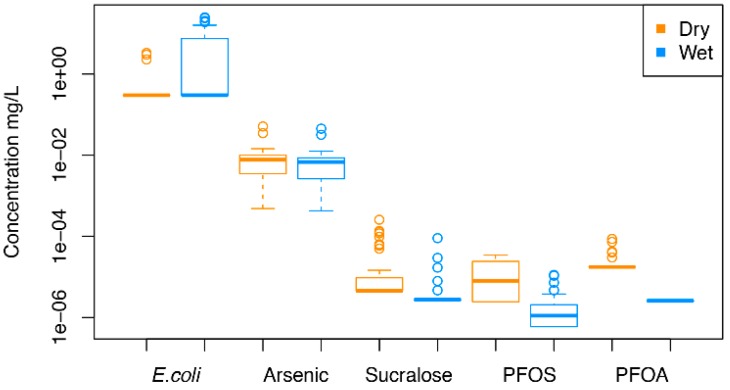
Contaminants that were significantly different by season, *E.coli*, sucralose, PFOS, and PFOA *p*-value ≤ 0.0001; arsenic *p*-value = 0.0004. PFOA: perfluorooctanoic acid; PFOS: perfluorooctane sulfate.

Water samples collected from private wells in this study had detectable levels (>0.03 CFU/100 mL) of *E. coli* in 7.5% and 48% of the dry and wet season samples, respectively (Table 3). There was a significant difference in detection frequency of *E. coli* between seasons, with *E. coli* detected in a greater number of wet season samples than dry season samples (McNemar’s *p*-value = 0.0004) (Figure 1).

Overall, the well water samples in this study contained detectable concentrations of the following inorganic contaminants: arsenic, cadmium, chromium, copper, lead, mercury, and nitrate (Table 3). Arsenic, nitrate, and *E. coli* exceeded U.S. EPA regulatory standards (MCL 0.01 mg/L, 10 mg/L, and 1 CFU/100 mL, respectively). During the dry season, the concentrations of arsenic and nitrate were both in exceedance of the MCL in 27.5% of the samples. The concentration of arsenic in the wet season was above the MCL in 20.0% of the samples, and the concentration of nitrate was above the MCL in 22.5% of the samples (Table 3). Arsenic concentrations were significantly higher in the dry season (Wilcoxon signed-rank *p*-value < 0.0001); all other inorganic contaminants were not statistically different between seasons.

PFOS and PFOA were both detected in dry season samples; PFOA was not detected during the wet season. Their combined concentrations exceed the 2016 U.S. EPA Public Health Advisory (PHA) level in three samples during the wet season (Table 3) [38]. There was a significant difference in PFOS concentrations between the dry and wet seasons, with levels higher in the dry seasons (Wilcoxon signed-rank *p*-value < 0.0001). There was a significant difference in detection frequency of sucralose between seasons, with sucralose detected in a greater number of dry season samples (10/40) than wet season samples (5/40) (McNemar’s *p*-value ≤ 0.0001).

## 4. Discussion

The levels (ND–25 CFU/100 mL) of *E. coli* in water samples collected from private wells in this study are consistent with what has been reported in other studies [2,12,18,19,20]. *E. coli* was detected in a greater number of wet season samples than dry season samples (McNemar’s *p*-value = 0.0004). While the private wells sampled in this study are located in a rural community, the community is within 20 miles (32.2 km) of the metropolitan cities of Nogales, Mexico, and Nogales, Arizona. Periods of heavy precipitation can result in sanitary sewer overflows in both Nogales, Mexico, and Nogales, Arizona, that lead to microbial contaminants affecting the surface waters along the Santa Cruz River [24,39,40]. The interaction between surface water and groundwater can result in microbial contaminants affecting well water, especially for sites in close proximity to the affected surface water [31]. In private well water, *E. coli* contamination has been attributed to proximity to livestock, agriculture and storm water runoff, and human sewage treatment systems (e.g., septic tanks and wastewater treatment plants) [2,12,20]. 

Arsenic, nitrate, and *E. coli* were the only contaminants tested that exceeded U.S. EPA regulatory standards (MCL 0.01 mg/L, 10 mg/L, and 1 CFU/100 mL, respectively). During the dry and wet season, arsenic was in exceedance of the MCL in 27.5% and 20.0% of the samples, respectively. Nitrate was above the MCL in 27.5% and 22.5% of the samples, respectively (Table 3). These percentages are higher than what was reported in a national study, where arsenic was above the MCL in 6.8% and above the nitrate MCL in 4.4% of the wells sampled [9]. Only arsenic was significantly different between seasons (Wilcoxon signed-rank *p*-value < 0.0001), with concentrations higher in the dry season. The arsenic concentrations in this study ranged from 4.84 × 10^−4^ to 5.08 × 10^−2^ mg/L. These concentrations are within the range of arsenic concentrations found in other areas of Arizona, 6.0 × 10^−4^ to 4.8 × 10^−2^ mg/L [11,41,42]. Arsenic is a naturally occurring inorganic contaminant that can be found in many areas of Arizona. Additionally, it is one of the contaminants that has been previously measured in effluent continuously discharged from the NIWTP [39,43]. Both of these sources likely have a greater influence on well and river water quality during the dry season. The seasonal variation of arsenic has been previously reported to be dependent on the local recharge, with some reporting higher levels in the wet season [44], and others in the dry season [45]. 

Although inorganic contaminants (e.g., arsenic, cadmium, chromium, copper, lead, mercury, and nitrate) are frequently detected in private well water, it is not always clear if their presence is due to naturally occurring processes or anthropogenic contamination [3,5,8,11,15,16,46]. In particular, multiple studies have reported private well water containing naturally occurring arsenic concentrations above the U.S. EPA MCL [8,10,11,15,16]. While the arsenic found in these wells is often attributed to the natural geology, processes such as the installation of new wells and exposed mine tailings from mining activities have been discussed as anthropogenic sources that can lead to increased arsenic concentrations in well water [8,15]. A known source of nitrate in well water is fertilizer from agricultural land water runoff and livestock facilities [4,12,47]. The private wells sampled in our study are located in a rural community in close proximity to land designated as agricultural and rangeland.

Before its expansion in 1989, the NIWTP was discharging effluent with high levels of mercury. It was presumed that mercury was originating from nearby industrial businesses [21]. The mercury levels reported in this study are similar to those reported as background levels in other private well studies [48]. In the wet season, *E. coli* was positively correlated with mercury (ρ = 0.34, *p*-value < 0.05) (Table 4). 

Mercury and arsenic were significantly correlated in the dry season and wet season (ρ = 0.87, *p*-value < 0.0001; ρ = 0.61, *p*-value < 0.0001, respectively). In the dry season, mercury was significantly correlated with lead (ρ = 0.62, *p*-value < 0.0001), PFOA (ρ = 0.53, *p*-value < 0.01), and PFOS (ρ = 0.32, *p*-value < 0.05) (Table 4). These dry season correlations could signify a similar source, and as concentrations of mercury vary only slightly across seasons, this might indicate that levels are likely related to the local geology, which would have less of an influence during the wet season. 

Sources of contaminants in well water can differ, and during periods of heavy precipitation urban storm water runoff can contribute to these differences [49]. Lead and copper were correlated in the wet season (ρ = 0.53, *p*-value < 0.0001). Copper and chromium were negatively correlated in the wet season (ρ = −0.32, *p*-value < 0.05), and chromium was positively correlated with arsenic (ρ = 0.40, *p*-value < 0.01). Plumbing, specifically if copper pipes or lead solder were used, could be a potential common source of copper and lead that has been reported in well water [23,50]. However, this is not likely in our study, as the taps were flushed for two minutes before sample collection to reduce the influence of water that had been stagnant in the plumbing. Additionally, both lead and copper are naturally occurring elements present in the Patagonia mountains near this study’s location [51], thus local geology may also be an important common source for lead and copper. 

Only three samples in the wet season exceed the 2016 U.S. EPA Public Health Advisory (PHA) for PFOA/PFOS (Table 3) [38]. The concentration of PFOS detected in this study (8.44 × 10^−7^ to 3.47 × 10^−5^ mg/L) is comparable to reported concentrations in other private well water studies, 2.4 × 10^−7^ and 8.35 × 10^−3^ mg/L [17,52]. PFOA was detected in more dry season samples than wet season samples (McNemar’s *p*-value < 0.0001) (Table 3). PFOA concentrations in this study (3.64 × 10^−6^ to 8.66 × 10^−5^ mg/L) are comparable to what has been previously detected in private well water, 6 × 10^−6^ to 1.33 × 10^−2^ mg/L [13,52]. In the dry season, PFOA was significantly correlated with arsenic (ρ = 0.42, *p*-value < 0.01), mercury (ρ = 0.53, *p*-value < 0.01), and lead (ρ = 0.46, *p*-value < 0.01). While many of the inorganic and microbial contaminants may have both natural and anthropogenic sources, perfluorinated chemicals, such as PFOS and PFOA, were previously produced by industrial companies for the manufacturing of Scotchgard™, Teflon®, carpets, and upholstering and coating additives [53,54,55].

Beginning in 2000, companies began to phase out PFOS and PFOA compounds, however, they continue to be detected in various environmental media, as they are highly persistent in the environment [56], and likely represent some end products from precursors of perfluoroalkyl carboxylic and sulfonic acids [57]. PFOS and PFOA have been simultaneously detected in effluent, surface waters, and well water samples. As PFOS and PFOA were incorporated into the same products, it is common to find these two contaminants in the environment together [22,58,59,60]. PFOS and PFOA were not only detected in this present study, but were significantly correlated with each other (dry season, ρ = 0.45, *p*-value < 0.01), a consistency reported in a previous study [61]. The lack of correlation between PFOA and PFOS in the wet season might indicate that while these man-made industrial chemicals may arise from similar sources (e.g., septic systems, biosolids and effluent from wastewater treatment plants), PFOA concentrations are low and vulnerable to urban and agricultural water runoff [17,22]. 

Unlike *E.coli*, PFOA, PFOS, and the inorganic contaminants detected in private well water that can have multiple sources, sucralose can only come from human waste. Sucralose is a commonly used artificial sweetener (e.g., Splenda) that is excreted by humans without any degradation during the digestion process. It is persistent in the environment, and its presence is most likely attributable to human waste, which can enter the environment as wastewater [62]. Currently, there is not a PHA level for sucralose, yet we chose to monitor it in private wells to determine if their water quality was potentially impacted by human waste. Sucralose has also been monitored and detected in the Santa Cruz River (the suspected source is the NIWTP), indicating that infiltration of sucralose from the river surface waters and human waste to well water is likely occurring [62]. Sucralose in the drinking water supply has been previously reported in levels ranging from 4.80 × 10^−5^ to 2.40 × 10^−3^ mg/L, which are within the levels found in our present study (6.05 × 10^−6^ to 2.57 × 10^−4^ mg/L) [63]. In this study, sucralose was detected in a higher number of dry season samples (25%) and *E. coli* was found in a greater number of wet season samples (48%), indicating different sources and seasonal trends. This might be due to sucralose’s persistence in the environment, and during the wet season, storm water runoff events could lead to dilution of sucralose in the well water. The lack of correlation between sucralose and *E.coli* likely indicates that they arise from different sources. While *E. coli* is not always correlated with pathogens or illness, its presence may indicate that there is contamination from animal and/or human feces and that there may also be enteric pathogens present in the water [64,65]. While both sucralose and *E.coli* likely arise from effluent discharged from septic tanks or NIWTP during the dry season, the greater concentration and presence of *E.coli* in the wet season is likely from additional input from animal feces in agricultural water runoff [66]. Additionally, sucralose was positively correlated with PFOS (wet season, ρ = 0.54, *p*-value < 0.0001), which may indicate similar sources, such as septic tank leakage or NIWTP. 

The Santa Cruz River in Arizona is 184 miles (296.1 km) long [24], and the wells sampled were within a 20-mile (32.2-km) stretch between Tubac, AZ, and Nogales, AZ. Future studies should expand the sampling location and assess if distance to the Santa Cruz River is associated with contamination. Additionally, there is a lack of information on factors that affect well water quality, such as well depth, water table depth, proximity to sewer pipelines, and septic tank usage. While a survey was administered to participants to identify this information, the majority (91%) of the participants answered ‘Do Not Know’ on the survey. Additionally, the four well water samples collected from the wellhead and not the kitchen sink were included in the data analysis. A sensitivity analysis was conducted where these four samples were excluded, however, this did not change the conclusion of the results. When the samples were analyzed for PFOS and PFOA in 2013 and 2014, the PHA levels were 200 ppt and 400 ppt, respectively. The detected PFOS and PFOA levels in this study were all below their respective PHA levels in 2013 and 2014. In 2016, the PHA level were reduced to a combined PFOS/PFOA Public Health Advisory of 70 ppt; as such, our study PFOS/PFOA levels now exceed the 2016 PHA for PFOA/PFOS for three samples in the wet season. 

## 5. Conclusions

The results of our study indicate that some private wells sampled in the Santa Cruz River area are contaminated with contaminants of public health concern, especially arsenic, nitrate, and *E. coli*. The wells are potentially contaminated by human waste, as indicated by the presence of sucralose. Due to the interactions between groundwater and surface water, with precipitation playing a key role in groundwater recharge, it appears that well water quality is being affected by multiple sources, such as local geology, human waste, the NIWTP, and urban and/or agricultural storm water runoff. Currently, the world is experiencing changing weather patterns, and it is predicted that the Southwestern United States will become hotter and drier [67], which could likely result in more effluent-driven rivers in time impacting local groundwater. While government agencies do not currently oversee well water quality, there should be efforts to provide necessary information to educate well owners about how to determine the quality of their well water. Governmental agencies, such as the U.S. Centers for Disease Control and Prevention, have established efforts to raise awareness and distribute educational information [68]. The American Academy of Pediatrics also recommends regular testing of well water and encourages that governmental agencies provide free or low-cost testing options for families [69]. Despite these efforts, there is a need to provide additional resources to well owners who do not have the ability or knowledge to test their well water [70]. Well owners whose water exceeds the MCL for regulated contaminants should consider using point-of-use filters or treatments such as reverse osmosis or chlorine tablets to improve their water quality. The treatment most appropriate for each well will depend on what contaminants are impacting the well water. 

## Figures and Tables

**Table 1:** Total precipitation by season from 2010 to 2014 in Nogales, AZ.

Year and Season	Total Precipitation (cm)	Percent of Total Precipitation that Year (%)
2010		
Dry Season^+^	1.27	4
Wet Season^++^	16.3	47
2011		
Dry Season	0.20	1
Wet Season	16.08	69
2012		
Dry Season	2.34	6
Wet Season	20.14	56
2013		
Dry Season	2.03	6
Wet Season	19.61	53
2014		
Dry Season	0.05	<1
Wet Season	25.35	55

Data collected from the weather underground using the weather station near Nogales, AZ, airport. ^+^ Dry season months (April, May, June); ^++^ Wet season months (July, August).

**Table 2:** Water quality measurements in well water by season.

Measurement	Season	Minimum	Median	Maximum	NSDWRs
**Temperature (°C)**					-
	**Dry**	20.5	25.0	32.4	
	**Wet**	21.0	25.3	29.3	
**TDS (mg/L)**					500
	**Dry**	170	273	490	
	**Wet**	35.3	275	478	
**pH**					6.5-8.5
	**Dry**	6.89	7.20	8.30	
	**Wet**	7.04	7.22	7.79	
**Conductivity (µs/cm)**					-
	**Dry**	340	546	977	
	**Wet**	258	587	958	

TDS; Total Dissolved Solids; NSDWRs: U.S. National Secondary Drinking Water Regulations.

**Table 3:** Summary of microbial, inorganic, and organic contaminant concentrations by season.

Analytes CFU/100ml or mg/L	Detection Frequency *n* (%)	Min	Mean	Median	Max	MCL or PHA^3^	% >MCL or PHA
*E. coli* ^1,***^						1	
Dry	3/40 (7.5)	ND	4.3	ND	25		7.5
Wet	19/40 (48)	ND	ND	ND	3.3		42.5
Arsenic ^2,*^						0.01	
Dry	40/40 (100)	4.84 × 10^−4^	9.60 × 10^−3^	7.71 × 10^−3^	5.08 × 10^−2^		27.5
Wet	40/40 (100)	4.24 × 10^−4^	8.39 × 10^−3^	6.78 × 10^−3^	4.53 × 10^−2^		20.0
Cadmium						0.005	
Dry	18/40 (45)	ND	1.16 × 10^−5^	ND	2.38 × 10^−4^		0
Wet	14/40 (35)	ND	1.17 × 10^−5^	ND	1.06 × 10^−4^		0
Chromium						0.1	
Dry	20/40 (50)	ND	1.83 × 10^−4^	ND	1.18 × 10^−3^		0
Wet	32/40 (80)	ND	2.40 × 10^−4^	9.68 × 10^−5^	1.10 × 10^−3^		0
Copper						1.3	
Dry	40/40 (100)	1.60 x 10^−5^	1.53 × 10^−2^	1.91 × 10^−3^	1.23 × 10^−1^		0
Wet	40/40 (100)	7.22 x 10^−4^	3.90 × 10^−2^	6.75 × 10^−3^	2.18 × 10^−1^		0
Lead						0.015	
Dry	34/40 (85)	ND	1.51 × 10^−4^	2.5 × 10^−5^	1.40 × 10^−3^		0
Wet	33/40 (82)	ND	1.66 × 10^−4^	7.6 × 10^−5^	1.25 × 10^−3^		0
Mercury						0.002	
Dry	31/40 (77)	ND	8.96 × 10^−5^	6.45 × 10^−6^	1.57 × 10^−3^		0
Wet	30/40 (75)	ND	5.30 × 10^−5^	1.31 × 10^−5^	1.59 × 10^−3^		0
Nitrate						10	
Dry	40/40(100)	3.10 x 10^−1^	9.35 ×10^+0^	5.92 × 10^+0^	5.24 × 10^+1^		27.5
Wet	40/40 (100)	3.10 x 10^−1^	9.18 ×10^+0^	4.55 × 10^+0^	5.25 × 10^+1^		22.5
PFOS ^2,**^						NA	
Dry	19/40 (47)	ND	1.20 × 10^−5^	7.98 × 10^−6^	3.47 × 10^−5^		NA
Wet	22/40 (55)	ND	2.07 × 10^−6^	1.11 × 10^−6^	1.12 × 10^−5^		NA
PFOA ^1,**^						NA	
Dry	5/40 (12.5)	ND	ND	ND	8.66 × 10^−5^		NA
Wet	0/40 (0)	ND	ND	ND	ND		NA
PFOA/S ^3,**^						7.0 x 10^−5^	
Dry	24/40 (60)	ND	3.64 × 10^−5^	2.62 × 10^−5^	1.16 × 10^−4^		0
Wet	22/40 (55)	ND	4.68 × 10^−6^	3.74 × 10^−6^	1.38 × 10^−5^		7.5
Sucralose ^1,**^						NA	
Dry	10/40 (25)	ND	2.71 × 10^−5^	ND	2.57 × 10^−4^		NA
Wet	5/40 (12.5)	ND	6.13 × 10^−6^	ND	9.03 × 10^−5^		NA

^1^ McNemar’s test performed on *E. coli*, Cd, PFOA, and sucralose ^2^ Wilcoxon signed-rank test performed on As, Cr, Cu, Pb, Hg, $NO_{3}^{-}$, ^3^ The Maximum Contaminant Level (MCL) is the highest level of a contaminant that is allowed in drinking water under the National Primary Drinking Water Regulations (NPDWR). The Public Health Advisories (PHA) are non-enforceable and currently used to provide technical information. The 2016 PHA is for the combined PFOS and PFOA concentration. ND: Not Detected; NA: Not Applicable, ** *p*-value ≤ 0.0001, * *p*-value = 0.0004.

**Table 4:** Summary of Spearman pairwise rank correlations by season.

**Dry**	***E. coli***	**As**	**Cd**	**Cr**	**Cu**	**Pb**	**Hg**	**NO_3_^−^**	**PFOS**	**PFOA**
**As**	−0.02									
**Cd**	−0.02	−0.02								
**Cr**	−0.02	0.11	0.20							
**Cu**	−0.13	−0.08	0.20	−0.16						
**Pb**	−0.12	0.44 ^**^	−0.06	−0.16	0.28					
**Hg**	−0.06	0.87 ^***^	−0.06	−0.12	0.01	0.62 ^***^				
**NO_3_^−^**	−0.17	−0.19	0.06	−0.21	−0.23	−0.19	−0.20			
**PFOS**	0.17	0.16	−0.18	−0.12	−0.10	0.17	0.32 ^*^	−0.18		
**PFOA^†^**	−0.10	0.42 ^**^	−0.10	−0.12	0.04	0.46 ^**^	0.53 ^**^	−0.21	0.45 ^**^	
**Sucralose**	0.09	−0.04	0.02	−0.24	0.27	−0.13	−0.11	−0.19	−0.01	−0.14
**Wet**	***E. coli***	**As**	**Cd**	**Cr**	**Cu**	**Pb**	**Hg**	**NO_3_^−^**	**PFOS**	**PFOA**
**As**	0.09									
**Cd**	0.07	0.10								
**Cr**	−0.01	0.40 ^**^	−0.08							
**Cu**	−0.28	−0.21	−0.05	−0.32 ^*^						
**Pb**	−0.22	−0.10	0.24	−0.21	0.53 ^***^					
**Hg**	0.34 ^*^	0.61 ^***^	−0.08	−0.09	−0.10	−0.07				
**NO_3_^−^**	0.18	−0.21	0.26	−0.17	−0.27	−0.19	−0.14			
**PFOS**	−0.14	−0.01	0.01	−0.24	0.17	0.06	−0.08	−0.27		
**Sucralose**	0.15	−0.05	−0.03	−0.12	0.06	−0.12	−0.04	−0.15	0.54 ^***^	

* Denotes significance *p* < 0.05; ** Denotes significance *p* < 0.01; *** Denotes significance *p* < 0.0001; ^†^ PFOA was only detected in the dry season and thus not included in pairwise rank correlations.
